# An Ecological Momentary Assessment Smartphone App for High-Risk HIV Populations: Development and Usability Study

**DOI:** 10.2196/85108

**Published:** 2026-05-01

**Authors:** Archana Krishnan, Pallavi Khurana, Alexandra R Stankus, Samy J Galvez, Jorge Sanchez, Frederick L Altice

**Affiliations:** 1 Department of Communication, University at Albany, State University of New York, Social Science 351, 1400 Washington Avenue, Albany, NY, 12222, United States, 1 518-442-3239, 1 518-442-3884; 2 Department of Internal Medicine, Section of Infectious Diseases, School of Medicine, Yale University, New Haven, CT, United States; 3 Centro de Investigaciones Tecnológicas, Biomédicas y Medioambientales, Universidad Nacional Mayor de San Marcos, Lima, Peru

**Keywords:** ecological momentary assessment, mHealth, usability testing, smartphone, Peru, people with HIV, men who have sex with men, antiretroviral therapy, adherence, feasibility and acceptability

## Abstract

**Background:**

HIV incidence has continued to increase among men who have sex with men (MSM) in Peru, despite intervention efforts. Addressing stigma, risky behaviors, and low medication adherence is key to reducing incidence rates. Ecological momentary assessment (EMA) allows for collection of discrete, real-time data on stigmatized, risky behaviors while reducing recall bias.

**Objective:**

The aim of this study was to develop and assess the usability of an EMA smartphone app among MSM with HIV in Peru, which tracks daily health risk behaviors to determine ease of use, usefulness, and satisfaction with the app.

**Methods:**

A mixed-method 3-phase study was conducted with 10 MSM with HIV, which included a usability test, 10-day field testing, and a debriefing focus group. Quantitative survey data and user analytics allowed for assessments of acceptability and user compliance. Qualitative interview and focus group data were thematically analyzed for in-depth assessments of user satisfaction.

**Results:**

Acceptability of the EMA app was high, with a mean usability rating of 6.4 of 7.0 (SD 0.62), indicating high user satisfaction, ease of use, and usefulness. A 10-day field test demonstrated a high average compliance rate of 93% (93/100), which suggests high feasibility of the app for daily tracking of health risk behaviors among MSM with HIV. Interview and focus group findings indicated that the app was navigable, time-efficient, and holds promise for long-term use, particularly with the inclusion of daily reminders and incentives for prolonged use.

**Conclusions:**

EMA apps can provide valuable real-time data while protecting users’ privacy. This formative work lays the foundation for future larger-scale EMAs of substance use and sexual risk behaviors among high-risk HIV populations, and for the development of just-in-time interventions to address stigma, improve medication adherence, and reduce risky behaviors.

## Introduction

The HIV epidemic in Peru—an archetypal middle-income country—is concentrated among men who have sex with men (MSM) [1-3]. HIV prevalence in this group is 10% to 22% [4,5], compared with less than 1% in the general population [6]. The HIV cascade is similarly concerning, with suboptimal levels of retention in care and use of antiretroviral therapy (ART), with a viral suppression rate of just 12% [2]. This has resulted in Peru being one of the few countries globally, since 2014, where HIV incidence continues to increase and HIV-related mortality has plateaued [7].

Despite a number of evidence-based HIV prevention and treatment strategies implemented by the Peruvian Ministry of Health, intervening with MSM in Peru has been challenging due to high levels of stigma and discrimination [8]. Same-sex behavior and HIV status are both stigmatized in Peruvian social and medical settings, and they are associated with psychological distress, risky sex, and suboptimal adherence to ART [9-11], all of which exacerbate health disparities. High drug and alcohol use in this population [12,13] further undermine adherence to ART [11] and are associated with increased sexual risk-taking [14,15]. This underscores the importance of providing efficacious HIV risk-reduction interventions for MSM that address stigma and substance use challenges.

Despite the many studies that show a significant relationship between substance use and risky sex, this relationship is not uniformly supported [16-18]. The conflicting findings are likely due to methodological limitations of retrospective data collection techniques, such as timeline follow-back, that are affected by recall bias, and prospective techniques, such as diary entries, that have low reliability [19,20]. A review of event-level research on substance use and sexual risk behavior showed that most studies were cross-sectional, employing either timeline follow-back or prospective daily diary entries, methods that have recall and social desirability bias [21]. There is thus a need for more robust event-level methodologies, such as ecological momentary assessment (EMA), which can capture real-time data on risk behaviors and pertinent contextual factors.

EMA refers to a collection of assessment techniques that includes behavioral observations, self-monitoring, and ambulatory physiological monitoring to measure behaviors, activities, or event-level data in real-world contexts and in real time [22-24]. EMA studies involve recurring assessments, albeit with varying degrees of intensity [25,26]. Since EMA occurs in real time or near real time and in one’s natural environment, recall bias is markedly reduced and ecological validity is strengthened [27]. Substance use behaviors, being discrete and episodic, lend themselves well to event-level assessments [20]. Theories of drug use highlight the importance of internal cues, situational factors, and social context, all of which cannot be replicated in experimental settings and are subject to recall bias in retrospective studies. EMA is therefore ideally placed to examine substance use, as data are recorded on an event-level basis using short-term recall [23,28]. This method, however, has not been leveraged with MSM with HIV to examine concurrent substance use and risky sex in South America.

With the ubiquity of mobile technology, mobile phones can be used to conduct EMA efficiently [29,30]. They can (1) increase participants’ privacy and enable ease of participation; (2) upload data in real time via wireless networks, preventing data loss; and (3) provide automatic time-stamping, geocoded data, and metadata to monitor participant compliance [31]. Studies have shown that smartphones are feasible for collecting substance use data among HIV-positive individuals and MSM [32,33], and that HIV-positive Peruvian MSM report high acceptability of technology-based studies to collect health behavior data [34]. To date, however, there is no published research on the development or use of smartphone-based EMA for examining health behaviors among key HIV populations—such as MSM and transgender women—in South America.

Effective interventions, especially just-in-time (JIT) interventions, require rigorous data on the concurrency of risk behaviors so that they can be correctly targeted. To achieve this long-term goal, the first step is to develop a smartphone-based EMA app to collect event-level data on substance use and sexual risk behaviors among MSM with HIV and conduct usability testing with this target population. This is the scope of the current study.

We developed an EMA smartphone app in English and Spanish, designed specifically to collect daily data on substance use, sexual risk behaviors, mood, and behavioral intention to engage in future risk behaviors. This formative work lays the foundation for a larger-scale EMA study of substance use and sexual risk behaviors among MSM with HIV.

## Methods

This study involved developing and testing a smartphone app to collect EMAs on daily substance use, unprotected sex, medication adherence, mood, and intent to engage in future risky behaviors. The EMA app, called PEMAA (Peru Ecological Momentary Assessment App), was developed on CommCare, an open-source app development platform. After alpha testing of the app, it was tested with a target population of 10 Peruvian MSM with HIV. The sample size was determined by referring to the usability testing literature, which recommends a sample of 3 to 5 [35], and using the cumulative binomial probability formula to estimate usability test sample size, which estimates 8 to be sufficient to discover usability issues [36]. Taking the upper limit, we supplemented this by 20% to account for dropouts during the 10-day field test, resulting in a sample size of 10.

Feasibility and acceptability testing of PEMAA used a three-phase, mixed methods protocol: (1) an in-person usability test of the app, (2) a 10-day field test, and (3) a debriefing focus group. During the in-person usability test, participants were audio-recorded while they downloaded and tested the app in front of the research team, after which they completed a quantitative assessment of app usability. Participants then field-tested the app for 10 days, during which daily reminder messages were sent to them to remind them to enter data on the app. Finally, at the end of field testing, a virtual focus group was convened to assess the participants’ collective experience of using PEMAA and obtain feedback for app improvement.

App assessment data collected across the 3 phases reflected the mixed methods nature of the design. The in-person usability test yielded both qualitative and quantitative data, the field test yielded data on user analytics, and the focus group yielded qualitative data on user experience and feedback. The study team was assisted by two research assistants: one aided in developing PEMAA, and the other helped conduct the 3 phases of the usability testing in Lima, Peru.

### Participants

Usability testing of PEMAA was conducted with 10 MSM with HIV who were recruited from the clinical site of the Centro de Investigaciones Tecnológicas, Biomédicas y Medioambientales (CITBM; in English, the Center for Technological, Biomedical, and Environmental Research) a nongovernmental research organization based in Lima. The CITBM is located on the main campus of the National University of San Marcos and provides health services to approximately 1600 MSM with HIV. Participants were eligible for this study if they were (1) aged 18 years or older; (2) born male; (3) self-reported HIV positive status; (4) self-reported any drug or alcohol use in the past 60 days; (5) self-reported any condomless sex in the past 60 days; and (6) currently owned an Android smartphone. The eligibility screening questions were assessed as binary items (yes/no).

### Ethical Considerations

Ethics approval for this study, including the study protocol and informed consent forms, was granted by the Clinical Trials Unit at the National University of San Marcos in Peru. Participants signed an informed consent form prior to the 3-phase usability test. They were informed about the voluntary nature of participation and could choose to withdraw from the study at any stage. All study materials were available in both English and Spanish. Participants were compensated with 25 Peruvian soles (US $7) for phase 1, 50 soles (US $14) for phase 2, and 25 soles (US $7) for the focus-group phase. They were also given bus tokens for traveling to the research site. To ensure confidentiality, the participants were instructed to enter mock data during the in-person usability testing and 10-day field testing, and all data and transcripts were deidentified prior to analysis.

### EMA App

PEMAA was developed on CommCare, an open-source app development platform. CommCare apps can only be downloaded on Android platforms; this is, however, not considered a limitation as Android is the most popular mobile platform in Peru, with 90.4% of market share [37]. For the purpose of field testing in Peru, we developed a bilingual (English and Spanish) version of PEMAA. Participants could use the app settings to toggle between the two versions depending on their preference. The daily survey contained 13 questions on alcohol and drug use, unprotected sex, ART adherence, mood, and the likelihood of engaging in future risky behaviors (the survey is available in Multimedia Appendix 1). The survey contained branching logic for specific questions on alcohol, drugs, and unprotected sex. For instance, if a participant responded positively to having consumed alcohol in the past 24 hours, the survey would branch to additional questions on drinking behavior before continuing to the rest of the survey.

**Figure 1.**
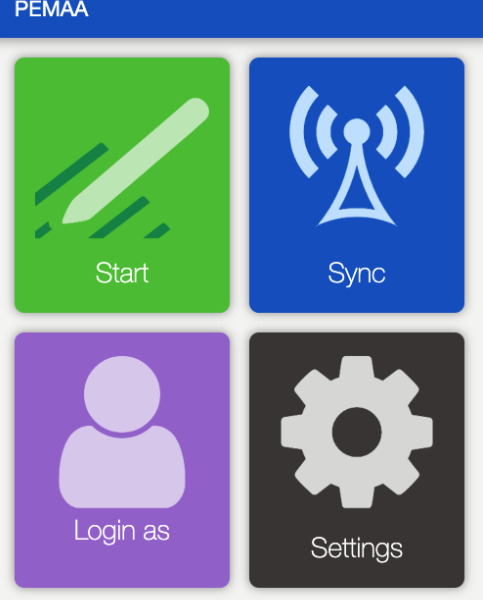
Screenshot of the PEMAA (Peru Ecological Momentary Assessment App) homepage.

**Figure 2.**
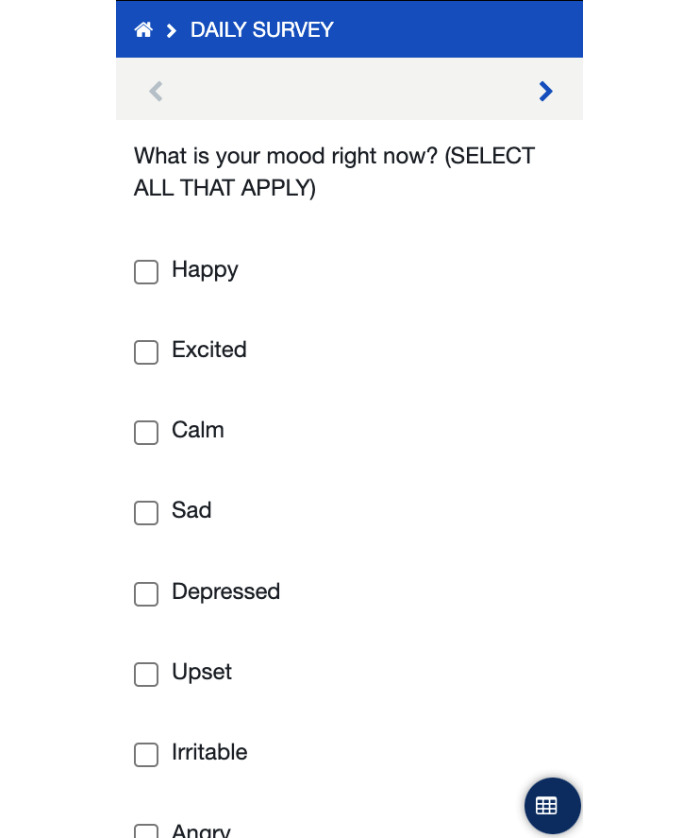
Screenshot of the PEMAA (Peru Ecological Momentary Assessment App) survey (English version).

As part of the app development process, initial versions of PEMAA were alpha tested by our research team. Alpha testing is an internal acceptance assessment and is a precursor to beta testing and/or field testing [38,39]. The aim is to identify potential technical barriers by checking accuracy, simulating user interaction, and incorporating initial feedback. The app design was kept purposefully simple and streamlined to ensure ease of use; for instance, the home screen had 4 distinct color-coded buttons (Figure 1). Clicking the green start button initiated the survey (Figure 2), with each question laid out on a separate page. After making changes and confirming the accuracy of the app, we created a version of PEMAA to be tested by a set of real-world users in Peru.

### mHealth App Usability Questionnaire

To quantitatively assess the usability of PEMAA, the mHealth App Usability Questionnaire (MAUQ) [40] was used. The MAUQ consists of 14 items scored on a 7-point Likert scale (1=“disagree” to 7=“agree”) and taps into dimensions of user satisfaction, ease of use, and usefulness. It has been used in multiple mobile health (mHealth) studies to appraise app usability [41,42]. In the present study, the MAUQ was translated into Spanish and back-translated to establish face validity [43], thus yielding the MAUQ-S scale (Table 1). The MAUQ-S is a unidimensional scale with acceptable internal consistency (Cronbach α=0.81).

**Table 1.** mHealth App Usability Questionnaire (MAUQ) in English and Spanish (MAUQ-S).

MAUQ items	MAUQ-S items
The app was easy to use.	La aplicación fue fácil de usar.
It was easy for me to learn to use the app.	Me fue fácil aprender a usar la aplicación.
The navigation was consistent when moving between screens.	La navegación fue consistente al navegar de una pantalla a otra.
The interface of the app allowed me to use all the functions (such as entering information and viewing information) offered by the app.	La interfaz de la aplicación me permitió usar todas las funciones de la aplicación (por ejemplo, ingresar información o ver información).
Whenever I made a mistake using the app, I could recover easily.	Al cometer algún error en la aplicación, me fue fácil corregirlo.
I like the interface of the app.	Me gusta la interfaz de la aplicación.
The information in the app was well organized, so I could easily find the information I needed.	La información en la aplicación está bien organizada, permitiéndome encontrar la información que necesito con facilidad.
The app adequately acknowledged and provided information to let me know the progress of my action.	La aplicación registró y me mostró adecuadamente progreso el de mi actividad.
I feel comfortable using this app in social settings.	Me siento cómodo al utilizar la aplicación en un lugar publico.
The amount of time involved in using this app has been convenient for me.	La cantidad de tiempo requerida para usar esta aplicación me ha sido conveniente.
I would use this app again.	Usaría esta aplicación de nuevo.
Overall, I am satisfied with this app.	En general estoy satisfecho/a/e con la aplicación.
The app would be useful for my health and well-being.	Esta aplicación me seria útil con mi salud y bienestar.
I could use the app even when the Internet connection was poor or not available.	Pude usar la aplicación aún cuando la conexión de internet estaba débil o no disponible.

### Procedure

#### Overview

Upon recruitment, participants were provided with a paper copy of the informed consent form and the instructions were read to them by the research assistant. After they provided informed consent, the participants were instructed to download PEMAA on their smartphones. A tutorial video (Multimedia Appendix 2) was created to help guide participants through downloading and installing PEMAA. To protect privacy and confidentiality, participants were instructed to enter mock data while they were observed interacting with the app, both during the in-person usability test and the 10-day field test. All informed consent forms and informational materials were written in both English and Spanish, although the Spanish version was primarily used on site in Peru.

#### Phase 1: Usability Testing

The usability test involved observing each participant using the app, which included various actions such as logging into the app with their assigned user credentials, initiating the EMA survey, reading and responding to each of the questions, and navigating the app. Participants were instructed to verbalize their actions and responses while interacting with the app and entering mock data. After each participant had tested the app, they were asked questions about the app, specifically its ease of use, navigability, and design; the suitability of its content; and their motivation to use it. They were also asked for their feedback on the EMA survey items. The verbalized usability test and the responses to the interviewer’s questions were recorded using a digital audio recorder. The audio files were later transcribed and translated into English before being analyzed to assess the user experience of PEMAA. At the end of this phase, participants completed the 14-item MAUQ-S. Finally, they were given instructions to field test PEMAA for 10 days.

#### Phase 2: Field Testing

To test the EMA app in real-world settings, each participant was instructed to fill out the survey on PEMAA and enter mock data for a period of 10 days. Mock data were preferred because the purpose of the study was to test the functionality of PEMAA and the acceptability of the EMA survey items, rather than to collect any personally identifiable health or behavioral data at this stage. However, every participant indicated their willingness to enter real data and did so during the 10-day field test; this was confirmed in the debriefing focus group. We mention this as an indicator of trust in PEMAA and a consideration for future deployment. Participants indicated their preferred time for receiving daily reminders to enter data on PEMAA; this was programmed accordingly for each participant.

#### Phase 3: Debriefing Focus Group

In the final phase, a focus group discussion was conducted on Zoom (Zoom Communications, Inc) to debrief participants and discuss their experience using PEMAA regarding ease of use, ability to enter data, intrusiveness of the EMA survey items, and overall satisfaction with the app and EMA survey. Other topics discussed in the focus group included privacy concerns and suggestions to improve the app, along with feedback on the sensitive nature of the survey items related to substance use and sexual risk behaviors. Finally, participants were asked to provide input on including stigma questions in the daily EMA survey. The focus group was conducted in the presence of a moderator and a note taker and was guided by a discussion protocol. The session started with the moderator noting that the proceedings would be recorded and encouraging participants to switch off their cameras and change the names on their profiles, so as to maintain a degree of anonymity. The discussion was audio-recorded, transcribed, and translated into English prior to being analyzed.

### Data Analysis

#### Quantitative Data

Quantitative data were generated in two ways: (1) the MAUQ-S scale was completed by participants during the in-person usability testing of PEMAA and (2) user analytic data were generated during the 10-day field test of PEMAA, which included date, time, and duration of app use, along with quantitative assessments of the survey items for each of the 10 days. The MAUQ-S and 10-day micro-longitudinal data were consolidated and analyzed using SPSS (version 28.0; IBM Corp). Despite participants’ disclosure of real data during the 10-day field test, we chose not to present those results for three reasons: (1) institutional review board approval was obtained primarily for the usability test and user experience of participants for PEMAA and did not cover behavioral health data; (2) the informed consent forms signed by the participants granted permission only to collect data on app usability and user experience; and (3) analysis of the micro-longitudinal health behavior data would not have been meaningful with such a small sample.

#### Qualitative Data

The usability testing interviews and focus group discussion were transcribed using a professional transcription service and then translated into English using DeepL Translator (DeepL GmbH) and Google Translate. Both sets of translations were consolidated and reviewed, and discrepancies were reconciled by a native Spanish speaker. The qualitative data were coded using NVivo (version 1.7; Lumivero) and analyzed using inductive thematic analysis, which is a qualitative method for identifying and analyzing patterns or themes.

Two coders followed the 6-step process outlined by Braun and Clarke [44]. First, they familiarized themselves with the data by conducting multiple readings of the transcript files. Second, they independently generated initial codes by systematically coding meaningful features within the dataset and collating relevant data for each code. Third, they conducted multiple rounds of analysis to search for themes by collating codes into potential themes. Fourth, they reviewed the themes and their relation to the codes and the entire dataset. Fifth, after further discussion, they defined and named the themes, thereby strengthening their understanding of the data captured by each theme. Discrepancies were reconciled through structured coder meetings using a shared codebook and documented memos, with final themes retained only when both coders agreed they were clearly supported by example quotations. Finally, they produced the report by selecting participants’ quotes from the data that clearly highlighted the essence of each theme.

With qualitative data, reliability is associated with consistency. A margin of variability in results is accepted as long as the method and epistemological logistics consistently generate ontologically similar data, even if these differ in richness and quality within similar dimensions [45]. It is important to note that although the same individuals participated in the usability testing group and the focus group, they did not have the same identification numbers in each group.

## Results

### User Analytics, Engagement, and App Acceptability

Results for user perceptions of PEMAA showed high acceptability (mean 6.4/7.0, SD 0.62) on the MAUQ-S scale. The MAUQ-S score ranged from 4.93 to 7.0 across participants and from 5.5 to 6.8 across all 14 items, thus indicating high user satisfaction, ease of use, and usefulness of the app. When considering user analytic data from the 10-day field test, average compliance was high at 93% (93 of 100 possible entries were made), despite the participant burden of entering sensitive health data daily. Half of the participants completed 100% of the daily surveys, while two participants had the lowest compliance at 80%.

**Figure 3.**
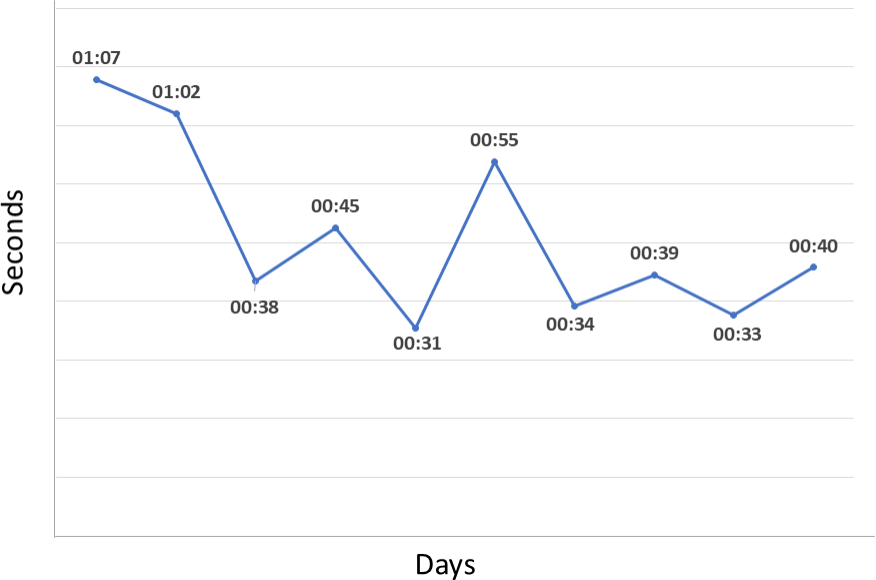
Average ecological momentary assessment survey completion time.

Considering usage data for EMA survey completion for each of the 10 days of field testing, participants completed the assessment in an average of 44 (SD 16) seconds. Seven participants completed the EMA survey in less than a minute each day of the 10-day testing period. Overall, the average time to completion was higher in the first 2 days (Figure 3) and decreased over the remaining days, indicating an experiential effect among study participants.

### Qualitative Findings

Themes and subthemes are summarized in Textbox 1.

Textbox 1. **Summary of themes and subthemes.**

- Usability (subthemes included ease of use and EMA [ecological momentary assessment] survey completion time)
- EMA survey questions
- User interface
- Willingness to use the app
- Recommendations (subthemes included incentives to use the app, the content of the questions, and daily reminders)

#### Theme 1: Usability

The literature defines usability as the extent to which users can use a product or system to achieve required goals with effectiveness, efficiency, and satisfaction in a specified context [46]. Users will find the product useful if it takes little time to complete a specific task, it is easy to understand, and it meets their expectations [47]. Participants described PEMAA as convenient with respect to usage and time. As part of the usability theme, we uncovered two subthemes: “ease of use and “EMA survey completion time.” This led users to use the app effectively to answer sensitive behavioral health questions.

Most participants reported that they found the app simple and easy to navigate. Some participants appreciated the intuitive design of the app, saying that it was easy to operate and made their experience seamless:

For my part, it has also been easy to use, I haven’t seen any complications.

[Participant 2; focus group]

Yes, it’s intuitive. it guides you by itself with every option you take. You don't necessarily have to do it on every button because the very use of it, the colors, maybe, or the actions you take makes you automatically understand what it’s for. Yeah, I'm fine with that. Easy to use.

[Participant 8; usability testing]

Participants also appreciated that accessing the app to complete the EMA survey was time-efficient. They reported that it did not take much time to answer questions and they could easily take the survey anywhere:

The time it takes to answer the questions, all good. It doesn't even take five minutes.

[Participant 7; focus group]

Yes, I could do it because it’s quite simple. In fact, it doesn't take any time at all. You can do it when you’re in the car, or while having breakfast—glancing at this or that—or when you’re taking something, especially medication. That same time you can do the survey.

[Participant 3; usability testing]

#### Theme 2: EMA Survey Questions

Participants commented that the survey questions were clear and concise, and several agreed that the questions were direct and easy to answer. For example, one participant mentioned that the questions were short and realistic:

They are not invasive questions; they are very direct and very easy to answer. Yes, the only thing I think is that the questions are well formulated, they are understood quite quickly.

[Participant 3; usability testing]

Furthermore, participants found the flow of questions logical, as they first assessed the individual’s mood and then their substance use and risky sexual behavior. This seemed to mitigate discomfort with some of the more pointed questions:

To me at least, I found it interesting that, first, it tries to address the mood of the person who is going to answer the questions before answering the directed questions themselves, because it asks you if you are happy, angry, how do you feel now, and then come the questions that are directed.

[Participant 1; focus group]

#### Theme 3: User Interface

Many participants commented on the user interface, stating that it was “attractive,” “interactive,” and “user-friendly.” A user interface is defined as what the user sees when interacting with the technology [48]. The appearance of smartphone apps has been found to impact the degree of user engagement and app use, with more appealing designs facilitating greater use [49]. An interface that is easily understood by users has also been suggested to impact mobile app usability by decreasing the cognitive effort required when using the app [50].

Easy. The interface is very user friendly. It’s easy to understand and connect.

[Participant 7; usability testing]

Although some participants reported finding the design to be attractive, others suggested that the design of the app could be more appealing. They suggested adding graphics or images would make the interface more “eye-catching,” and one participant suggested the addition of characters or avatars. Although most participants reported finding PEMAA easy to use, some issues were raised related to navigability. One participant suggested that enlarging the font and changing the color might make the app more navigable. Comments were also made that progression through the survey questions should be automatic rather than prompted by the user. Additionally, although the app was programmed to toggle between English and Spanish, the language option did not apply to the log-on page, which was in English by default; participants recommended that this option be made available.

I see that you have the option to change the language, which is important. Everything is in English in the settings, but I can change the language. Only the language changes for the questions, not for the information in the application.

[Participant 9; focus group]

#### Theme 4: Willingness to Use the App

One area which is under-researched in mHealth usability studies is the duration individuals are willing to participate in EMA studies and interventions [51]. When questioned about this, one participant noted that it would depend on the amount of time each individual has available. This was reflected in other participants’ responses, as some indicated they would use the app for an extended period of time, while another stated that approximately 2 weeks would be the maximum amount of time that they would adhere to using the app. Another participant said they would use the app on their own for a maximum of 30 days, but were willing to use it for a longer period with reminders to complete the surveys. Several participants agreed that reminders would be essential for prolonged use; for example,

With respect to filling out the questions, it depends on one’s time per se, because you have to take into account that some people also study or work, in my case. You also have to take into account the issue of reminders. For my part, it was difficult for me to fill them out, if it were not for the reminder sent to me by WhatsApp. Yes, I filled them out by myself, because I was resting or something, but if they wanted, for example, to fill it out for constant months, they would have to add the reminder through the same application.

[Participant 7; focus group]

Participants had been instructed to enter false data for the purpose of field-testing the app, but all ultimately said they entered real data. In response to their decision to input real data, participants explained that they were comfortable using real data as personally identifying information would not be collected. This finding is significant, as privacy and security have been found to be particularly important to users of health-related mobile apps [52] and may be of even greater importance to users with often-stigmatized conditions such as HIV. The quotes below show how participants were able to compartmentalize this issue and feel comfortable entering sensitive health data.

They’re not asking me for personal data, whether it’s names or exact identity. So there wouldn’t be any discomfort.

[Participant 2; usability testing]

If it’s for a health study or whatever, there’s no problem.

[Participant 6; usability testing]

#### Theme 5: Recommendations

Participants voluntarily provided multiple recommendations along with their feedback, which formed this overarching theme. Many of the recommendations were uncovered in the focus group, as by then, participants had 10 days of use experience with PEMAA. The 3 subthemes—incentives to use the app, the content of the questions, and daily reminders—indicated how participants visualized higher compliance and better engagement in using the app daily.

When asked what might motivate regular use of PEMAA to enter health data, participants suggested incentives such as discounts to local restaurants or saunas. Another participant pointed out that vouchers might be more motivating. One participant explained that an incentive could be offered after a certain number of days of survey completion.

For a number of days. If you complete a number of days you get a discount on a hamburger, Bembos, something like that. A Burger King, a KFC or a discount.

[Participant 1; focus group]

The EMA questions were phrased in a detailed way to avoid confusion; however, participants suggested that they could be shortened for efficiency, which has been suggested to be a key component in promoting usability [51]. Additionally, although participants reported that the questions were easy to answer, they said that responding to the same questions every day could become monotonous, suggesting that the questions might be paraphrased to read differently but collect the same information.

The surveys were very interesting, but what I would have liked is that the questions would have changed every day during the 10-day journey, each day would have been asked certain questions, but they were interesting and easy to answer.

[Participant 6; focus group]

One participant even suggested that additional questions could be included to collect more information, such as the frequency of drug and alcohol use or the reason for using drugs and alcohol.

Maybe you can have some more information. There may be something else, not intrusive, but maybe more information can be gathered. It could be perhaps caregiving, sexual relations. Even, the type of drugs that are used or how often alcohol or drugs are used, for what reasons, or it could be maybe for fun or maybe for addiction. I don't know if they're relevant, but it might be a little more informative.

[Participant 7; usability testing]

The key to compliance was attributed to the daily reminders participants received, prompting them to open PEMAA and complete the EMA. Many participants stated that daily reminders were important, especially if they were expected to fill out the surveys for a longer period. Without a reminder, one participant said 30 days would be the maximum amount of time they would be willing to use the app. Several participants expressed that they would prefer receiving push notifications from the app rather than through WhatsApp.

The reminder, that the application gives you the option to remind you, because it gave us reminders, I think, by a WhatsApp bot, but the ideal thing would be for the application itself to allow you to remember it, to send you an alert, “You have to fill it out.”

[Participant 1; focus group]

## Discussion

### Principal Findings

This was the first culturally specific EMA app designed to collect sensitive daily risk behavior data from MSM with HIV in Peru. Usability tests indicated high acceptability of the app, yielding a score of 6.4 out of 7.0 on the MAUQ-S scale. Most importantly, despite the burden of entering sensitive health data daily, compliance was 93%, with some participants achieving 100% compliance. The simplicity of PEMAA and ease of use in entering data likely influenced compliance. Feedback from the debriefing focus group reinforced these findings, indicating that participants perceived the app as simple, intuitive, and easy to navigate. They also highlighted that the survey questions were concise and direct, allowing for ease of completion. The logical flow of questions, beginning with mood assessments and then progressing to inquiries about substance use and sexual behaviors, appeared to reduce discomfort with more sensitive items. From a methodological point of view, starting the survey with mood questions before moving on to risk behaviors was intended to explore causation effects, as this structure may have encouraged more truthful responses while positively influencing compliance.

In EMA studies, compliance is a significant issue due to significant participant burden in entering event-level data; this is particularly stark when involving substance users. A recent meta-analysis showed that EMA compliance in studies with substance users had a pooled compliance rate of 75% across all studies, regardless of frequency or duration of assessments; this is lower than the recommended 80% compliance rate [53,54]. Low compliance is associated with missing data, which has a detrimental effect on statistical power and can skew inferences made on the basis of systematic bias [55]. Although we observed high completion rates during the 10-day field test, adherence over longer, more typical EMA durations of 30 to 60 days [56,57] may be lower, particularly outside a research context, where a lack of compensation can increase friction [58]. Longitudinal mHealth studies consistently show that participant burden, competing demands, and “survey fatigue” contribute to attrition and declining response rates over time, even when initial usability is high. In this context, participants’ suggestions to use incentives (eg, vouchers) should be interpreted as a pragmatic strategy to offset perceived burden and sustain engagement, rather than as a simple preference for rewards. Accordingly, mHealth implementation should prioritize burden-reducing strategies such as minimizing assessment items, tailoring prompt timing, and leveraging passive data (eg, user analytics) where appropriate. These strategies are particularly salient given that our field-test compliance for PEMAA likely benefited from simple app design and daily reminder prompts in addition to participant compensation.

We obtained valuable feedback on the acceptability of PEMAA, especially regarding app design and navigability. The app was designed to be simple, with intuitive navigation; however, recommendations were made on enhancing the appearance of the app via images, graphics, and color to avoid monotony. Although participants reported feeling comfortable answering sensitive questions, it was noted that some individuals might be embarrassed, and that therefore, the ability to skip questions should be included. Recommendations were also made to make progression faster by automatically progressing to subsequent pages upon response selection. Our team made this design decision to enable thoughtful selection of responses and to allow participants to change their answer before progressing. All of the above recommended changes can be easily made on the CommCare platform; however, one limitation of the platform is the inability to modify standardized options, such as changing language options on the app installation and log-on pages. Thus, although PEMAA is supported in both English and Spanish, and can potentially be adapted to multiple languages, the use of English on the log-on page by default likely poses a usability issue.

Most notable in this study was that despite being instructed to enter mock data during field testing, every respondent acknowledged entering genuine, real-world data. This behavior underscores the degree of trust participants placed in the smartphone app as well as their comfort in providing sensitive health information. Participants affirmed that perceived data security and anonymity fostered their willingness to provide sensitive health information. This finding is significant, as privacy and security have been found to be particularly important to users of health-related mobile apps [52] and may be of further importance to those experiencing stigma, such as MSM with HIV and other marginalized communities.

### Limitations

Within the context and value provided by this formative research, we must also address some limitations. While the sample size was justified for usability testing, the duration of the testing period was relatively short, meaning that long-term feasibility and sustained compliance were not evaluated. Furthermore, although participants reported being comfortable providing sensitive health information, social desirability bias cannot be ruled out. Despite having the ability to modify PEMAA based on participants’ recommendations, some technical recommendations, such as applying language options to the app log-on interface, are reliant on the CommCare development platform. Finally, although the rigor and trustworthiness of the qualitative findings were ensured through independent coding and iterative refinement, a numerical indicator of intercoder reliability was not calculated.

### Future Work

Having shown the feasibility and acceptability of PEMAA in the target audience of individuals in Peru who use substances, are at a high risk, and are HIV positive, the next step is to conduct a larger-scale EMA to (1) assess compliance over an extended period of time and (2) establish a temporal link between contextual factors like mood and stigma and substance use, sexual risk-taking, and medication adherence. In the focus group, we gauged the feasibility of adding stigma questions in the daily EMA; this was strongly supported by the study participants. Expanding the app to include questions on stigma and discrimination would help explain the complex interactions between substance use, sexual risk, and health behaviors. The long-term goal seeks to develop JIT interventions to improve ART adherence and reduce concurrent substance use and risky sex among MSM with HIV.

### Conclusion

This formative work demonstrates the usability and acceptability of a culture-specific smartphone-based EMA app to collect recurring event-level data on substance use and sexual risk behaviors among MSM with HIV. The findings show that trust, simplicity, and logical survey design contribute to app compliance and satisfaction. While certain design limitations must be addressed, the results lay an important foundation for scaling EMA use in behavioral health research, especially for marginalized and stigmatized populations. Ultimately, this work represents a critical step toward the development of JIT interventions designed to reduce risk behaviors and improve health outcomes in high-risk HIV populations.

## Supplementary material

10.2196/85108 Multimedia Appendix 1 EMA survey instrument in English and Spanish.

10.2196/85108 Multimedia Appendix 2 App installation video tutorial for users in Spanish.

## Abbreviations

ART: antiretroviral therapy
CITBM: Centro de Investigaciones Tecnológicas, Biomédicas y Medioambientales
EMA: ecological momentary assessment
JIT: just in time
MAUQ: mHealth App Usability Questionnaire
mHealth: mobile health
MSM: men who have sex with men
PEMAA: Peru Ecological Momentary Assessment App
SUD: substance use disorder
