# Supplementary material for: An Ecological Momentary Assessment Smartphone App for High-Risk HIV Populations: Development and Usability Study
Source: JMIR Form Res. 2026 May 1;10:e85108. doi: 10.2196/85108 (PMC13134826; doi:10.2196/85108)
Supplement: Multimedia Appendix 1 [file formative-v10-e85108-s001.pdf]

EMA Survey

1. What is your mood right now? (SELECT ALL THAT APPLY)
   - Happy
   - Excited
   - Calm
   - Sad
   - Depressed
   - Upset
   - Irritable
   - Angry
   - Ashamed

2. Did you take your HIV medicine in the past 24 hours?
   - No
   - Yes

3. Have you had any alcohol in the past 24 hours?
   - No
   - Yes

4. How much alcohol did you consume in the past 24 hours?
   A standard alcoholic beverage would be a bottle/can of beer, glass of wine, shot of vodka/rum/whiskey etc.
   - 1-2
   - 3-4
   - 5-6
   - 7-9
   - 10 or more

5. Have you taken any drugs in the past 24 hours?
   - No
   - Yes

6. What type of drugs did you take in the past 24 hours? (SELECT ALL THAT APPLY)
   - Cocaine
   - Amphetamines (Benzedrine, Dexedrine, Ritalin, Preludin, Methamphetamine, Speed, Ice, Crystal)
   - Special K (Ketamine)
   - Ecstasy (X, Molly, MDMA)
   - Gamma Hydroxybutyrate (G, Liquid ecstasy)

7. Did you have anal sex in the past 24 hours?
   - No
   - Yes

8. Did you use a condom for all anal sex you had?
   - No
   - Yes

9. Were you drunk or high during anal sex?
   - No
   - Yes

10. What was your mood before anal sex? (SELECT ALL THAT APPLY)
    - Happy
    - Excited
    - Calm
    - Sad
    - Depressed
    - Upset
    - Irritable
    - Angry
    - Ashamed

11. How likely are you to…?

Drink alcohol today:

1	2	3	4	5	6	7
Extremely unlikely	Very unlikely	Somewhat unlikely	Neutral	Somewhat likely	Very likely	Extremely likely

Use drugs today:

1	2	3	4	5	6	7
Extremely unlikely	Very unlikely	Somewhat unlikely	Neutral	Somewhat likely	Very likely	Extremely likely

Have anal sex today:

1	2	3	4	5	6	7
Extremely unlikely	Very unlikely	Somewhat unlikely	Neutral	Somewhat likely	Very likely	Extremely likely

Encuesta EMA

12. ¿Como te sientes en este momento? (ESCOGE TODAS LAS OPCIONES QUE SEAN APLICABLES)
    - Feliz
    - Emocionado
    - Tranquilo
    - Triste
    - Deprimido
    - Molesto
    - Irritable
    - Enojado
    - Avergonzado

13. ¿Tomaste tu medicina para el VIH en las ultimas 24 horas?
    - No
    - Si

14. ¿Has consumido alcohol en las ultimas 24 horas?
    - No
    - Si

15. ¿Cuanto alcohol consumiste en las ultimas 24 horas?
    Una bebida alcohólica estándar consiste de una botella o lata de cerveza, una copa de vino, o un shot de vodka/ron/wiski, etc.
    - 1-2
    - 3-4
    - 5-6
    - 7-9
    - 10 o mas

16. ¿Has consumido drogas en las ultimas 24 horas?
    - No
    - Si

17. ¿Que tipo de drogas consumiste en las ultimas 24 horas? (ESCOGE TODAS LAS OPCIONES QUE SEAN APLICABLES)
    - Cocaína
    - Anfetaminas (Benzedrine, Dexedrine, Ritalin, Preludin, Methamphetamine, Speed, Ice, Crystal)
    - Special K (Ketamina)
    - Ecstasy (X, Molly, MDMA)

- Gamma Hydroxybutyrate (G, Liquid ecstasy)

18. ¿Tuviste sexo anal en las ultimas 24 horas?
    - No
    - Si
19. ¿Usaste condón todo el tiempo durante el sexo anal?
    - No
    - Si

20. ¿Estabas borracho o bajo la influencia de las drogas mientras tenias sexo anal?
    - No
    - Si

21. ¿Como te sentías antes de tener sexo anal? (ESCOGE TODAS LAS OPCIONES QUE SEAN APLICABLES)
    - Feliz
    - Emocionado
    - Tranquilo
    - Triste
    - Deprimido
    - Molesto
    - Irritable
    - Enojado
    - Avergonzado

22. ¿Que tan probable es que…?

Bebas alcohol hoy:

1	2	3	4	5	6	7
Muy improbable						Muy probable

Consumas drogas hoy:

1	2	3	4	5	6	7
Muy improbable						Muy probable

Tengas sexo anal hoy:

1	2	3	4	5	6	7
Muy Improbable						Muy probable
